# Research on the influence of key structural parameters on piston secondary motion

**DOI:** 10.1038/s41598-021-98686-2

**Published:** 2021-09-27

**Authors:** Haixiang Yang, Jilin Lei, Xiwen Deng, Jun Wen, Zhigao Wen, Guofu Song, Rui Mo

**Affiliations:** 1 Yunnan Key Laboratory of Internal Combustion Engines, Kunming University of Science and Technology, Kunming, 650500 People’s Republic of China; 2 Yunnan Key Laboratory of Plateau Emission of Internal Combustion Engines, Kunming Yunnei Power CO., LTD, Kunming, 650200 People’s Republic of China; 3 Chengdu Galaxy Power CO., LTD, Chengdu, 610505 People’s Republic of China; 4 Kunming Yunnei Power CO., LTD, Kunming, 650200 People’s Republic of China

**Keywords:** Engineering, Mechanical engineering

## Abstract

Piston secondary motion not only influences the side knocking of piston and frictional loss, but also influence the in-cylinder oil consumption and gas blow-by. An inline four-cylinder common rail diesel engine was chosen as the research object. Dynamic simulation model of piston assembly was built based on the piston and cylinder liner temperature field test. The impacts of pinhole offset, liner clearance and piston skirt ovality on piston secondary motion were researched. Based on the surface response method, the influence of multiple factors on friction power loss and slapping energy is estimated. The results indicate that: in-cylinder stress condition of piston will change with its structural parameters, then the secondary motion of piston will be affected as a result. Pinhole offset, liner clearance, piston skirt ovality and the interaction of the latter two all have significant effects on the friction power loss, while the slapping energy is significantly affected by liner clearance. Therefore, the parameters can be designed based on the significance level to optimize the secondary motion characteristics of the piston.

## Introduction

The dynamic characteristics of the piston assembly have a great impact on the performance of the piston slapping energy, in-cylinder oil consumption and blow-by of the internal combustion engine. The force of the piston is complicated. It works under the combination of gas pressure, shaft reaction force, friction force, side force and other loads. In addition to reciprocating linear movement along the cylinder axis, it also performs lateral movement and rotation around the pin axis in the cylinder. These two movements are generally called the piston secondary motion^1–5^. The severe secondary movement is the main cause of wear of the piston rings and the slapping of the piston on the liner. The automotive industry has gradually improved the performance requirements of the internal combustion engine NVH (Noise, Vibration and Harshness). Piston slapping is an important part of the source of vibration and noise^6–8^. Therefore, the optimized design based on the key structural parameters of the piston secondary motion would reduce the vibration and noise caused by the piston slapping, the cylinder liner cavitation, friction loss of the piston assembly and oil consumption, etc.

In recent years, piston secondary motion characteristics have been a research hotspot. Bench test and simulated test research are the most direct way^6,9–11^. Research by Honda’s Hirotaka Murakami showed that when the piston leant on the ATS (anti-thrust side) during compression stroke, it rapidly moved back to the TS (thrust side) after reaching the maximum cylinder pressure at TDC (top dead centre). When the pinhole offset was on the TS, the slapping energy would be reduced^5^. Shah of the University of Auckland took an asymmetric half-piston model as the research object. Piston secondary motion and pinhole contact condition were analysed combined with tests. Elastohydrodynamic lubrication and the surface roughness at the hole-skirt boundary were also considered. Analysis factors included hole deformation caused by mechanical and thermal load as well as inertial load, the cylindricity and ovality of the piston^9^. Nobutaka Tsujiuchi of Doshisha University in Japan found that in a four-stroke engine, the TS of the piston collided with the cylinder liner three times in one working cycle, and the strongest collision occurred in the TDC of compression stroke^10^. Liu, T. of Shandong Binzhou Bohai Piston Co., LTD. used the maximum side force and friction loss of a high-strengthened diesel engine piston as the main evaluation indicators to optimize the piston skirt profile. Cylinder scuffing in the high temperature test was solved after the optimization^11^.

The reliability of theory and simulation model analysis based on multi-body dynamics has also been further improved^7,12–18^. N. Dolatabadi of Loughborough University applied the TET (targeted energy transfer) theory to the secondary motion of passively controlled pistons. A piston component model (stiffness-damping combination) based on the concept of nonlinear energy absorbers was established. It could reduce the secondary movement of the piston and reduce the slapping on the cylinder liner^7^. Guo, L. of Zhejiang University applied the dynamic slapping force of the secondary motion to the cylinder liner as a load boundary condition on the finite element model. The vibration response of the cylinder block under the piston slapping was calculated. In addition, simulation model was verified by bench test^12^. Guo, J. of Harbin Engineering University studied the relationship between the piston-cylinder clearance, the small head clearance of the connecting rod and the secondary motion of the piston. He carried out in-depth dynamic theory and simulation analysis, which were of significant theoretical meaning to optimize the selection of two clearance values and reduced the friction loss of piston assembly^13^. Wang, W. L. of North University of China built the finite element model of the piston considering the secondary motion of the piston. Dynamic calculations showed that the cylinder clearance is the most significant influence on the peak dynamic slapping force, followed by pinhole offset and the Y offset of the piston centre of gravity^14^.

The above and some other studies carried out relevant analysis on the test, simulation method or some influencing factors of the piston secondary motion. Nonetheless, there are few comprehensive studies on the influence of the piston structural parameters on the piston secondary motion and the multi-parameter interaction relationship. To this end, a dynamic simulation model of the piston assembly was built based on the surface temperature test of the piston and cylinder liner. The single factor effect of key structural parameters such as pinhole offset, cylinder clearance and skirt ovality on the piston secondary motion was studied respectively. Response surface methodology^19^ was adopted to analyse the interaction effects of three parameters on the piston secondary motion.

## Methods

### Research object

A piston assembly model of an inline four-cylinder common rail diesel engine was chosen as the research object. Part of the engine’s key parameters are listed in Table 1.

**Table 1:** Key parameters of the engine.

Parameter	Value
Bore (mm)	102
Stroke (mm)	115
Compression ratio	17:1
Displacement (L)	3.76
Combustion chamber structure	ω
Intake method	Turbocharging
Calibrated power (kW/r min^−1^)	85/2400
Peak torque (N m/r min^−1^)	360/1500
Connecting rod length (mm)	192

### Piston dynamic analysis

#### Piston kinematic analysis

**Figure 1:**
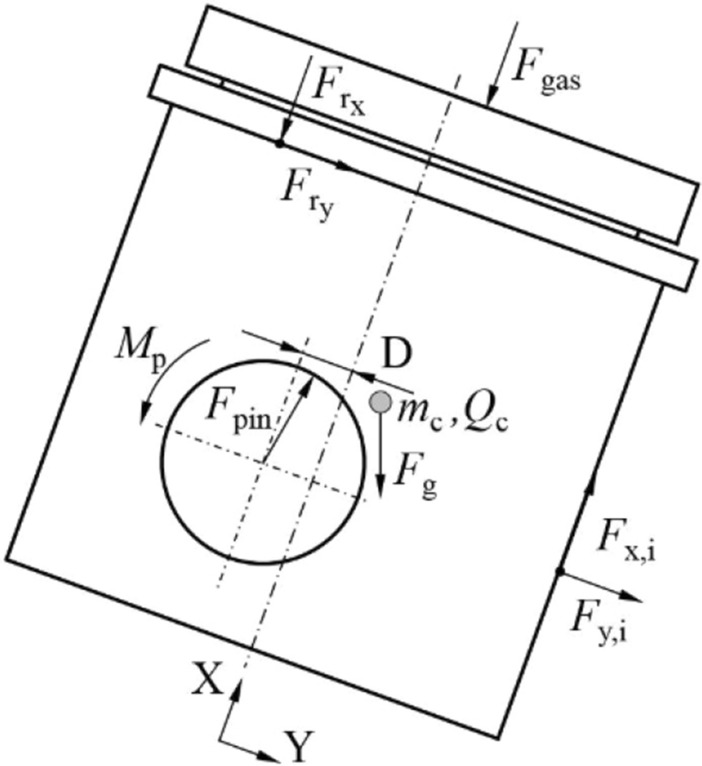
Piston kinematic analysis.

According to the principle of dynamic load balance^13,20–23^, the piston force analysis is shown in Fig. 1. The X direction is from the bottom to top along the axis of the piston, the Y direction is from the TS of the piston points to the ATS perpendicular to the X direction.

The piston dynamics equations in the two directions are shown in Eqs. (1)–(3).

Translation motion in X direction:

1 $$m_{c} \cdot \ddot{x}_{c} = \sum F_{x} = \mathop \sum \limits_{i = 1}^{2} F_{x,i} - F_{{g_{x} }} - F_{gas} - F_{{r_{x} }} + F_{{p_{x} }}$$

Translation motion in Y direction:

2 $$m_{c} \cdot \ddot{y}_{c} = \sum F_{y} = \mathop \sum \limits_{i = 1}^{2} F_{y,i} - F_{{g_{y} }} - F_{{r_{y} }} + F_{{p_{y} }}$$

Rotation around the axis of the piston pin:

3 $$Q_{c} \cdot \ddot{K}_{c} = \sum M = M_{c} + M_{g} + M_{gas} + M_{r} + M_{p}$$

where *m*_c_ (kg) is the mass of the piston. *Q*_c_ (kg·m^2^) is the moment of inertia of the piston around the piston pin. *K*_c_ is the angular acceleration of the piston around the piston pin. *F*_i_ (i = 1, 2) is the piston side force on the TS or ATS generated by contact with the liner. *F*_g_ is the piston gravity. *F*_gas_ is the in-cylinder gas pressure. *F*_r_ is the contact force between the rings and the piston. *F*_p_ is the force of the pin on the pinhole. *M*_c_ is the moment produced by the contact force between the piston and the liner. *M*_g_ is the moment around the piston pin caused by the gravity of the piston. *M*_gas_ is the moment produced by the in-cylinder gas pressure. *M*_r_ is the moment generated by the axial and radial forces of the piston ring. *M*_p_ is the friction moment produced by the contact force between the pinhole and the pin.

#### Piston assembly lubrication analysis

The Reynolds equation is used to describe the relationship between lubricating oil film pressure and thickness, and the Reynolds equation can be directly derived from the Navie-Stokes equation and the continuity equation as follows:

4 $$\frac{\partial }{\partial x}\left( {\frac{{h^{3} }}{12\eta } \cdot \frac{\partial p}{{\partial x}}} \right) + \frac{\partial }{\partial z}\left( {\frac{{h^{3} }}{12\eta } \cdot \frac{\partial p}{{\partial z}}} \right) = \frac{{u_{1} + u_{2} }}{2} \cdot \frac{\partial h}{{\partial x}} + \frac{\partial h}{{\partial t}}$$

where *u*_1_, *u*_2_ (m/s) are the velocity of the two contact surfaces. *p* (Pa) is the pressure of oil film. *h* is the thickness of oil film. $\eta$ (Pa·s) is the oil viscosity. This equation illustrates the relationship between oil film thickness, oil film pressure, relative speed, and oil viscosity in space x–z with time t.

**Figure 2:**
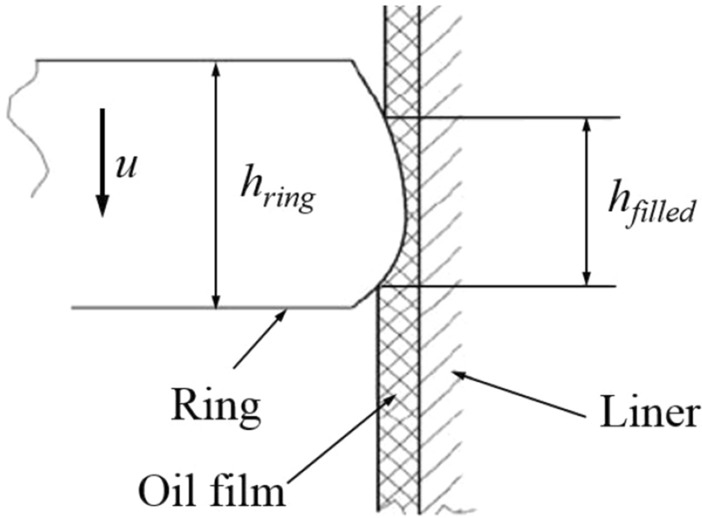
Oil filling rate.

In order to better reflect the cavitation that may occur in the actual movement process, the above-mentioned Reynolds Eq. (4) is extended with the oil filling rate. The oil filling rate is defined as:

5 $$\theta = \frac{{h_{filled} }}{{h_{ring} }}$$

where *θ* is the oil filling rate. *h*_*filled*_ is the height of lubricated area. *h*_*ring*_ is the height of the ring. Figure 2 illustrates the definition. When *θ* = 1, it can be considered that the oil is completely filled. While *θ* < 1, it means cavity area exists there. The oil filling rate of piston skirt-liner can also be defined in the same way.

After introducing the oil filling rate, the extended Reynolds equation is:

6 $$\frac{\partial }{\partial x}\left( {\frac{{h^{3} }}{12\eta } \cdot \frac{\partial p}{{\partial x}} \cdot \theta } \right) + \frac{\partial }{\partial z}\left( {\frac{{h^{3} }}{12\eta } \cdot \frac{\partial p}{{\partial z}} \cdot \theta } \right) = \frac{{u_{1} + u_{2} }}{2} \cdot \frac{\partial h}{{\partial x}} \cdot \theta + \frac{{u_{1} + u_{2} }}{2} \cdot \frac{\partial \theta }{{\partial x}} \cdot h + \frac{{\partial \left( {h\theta } \right)}}{\partial t}$$

Obviously, the secondary motion of the piston would significantly affect the lubrication effect between the piston assembly and the liner. The more significant the secondary motion characteristics of the piston, such as the tilting angle, radial displacement, and side impact force, the worse the uniformity of lubricating oil film thickness and pressure distribution on the contact surface. Dry friction is prone to occur at positions where the oil is insufficiently filled, which increases friction power loss.

### Temperature field test of piston and cylinder liner

**Figure 3:**
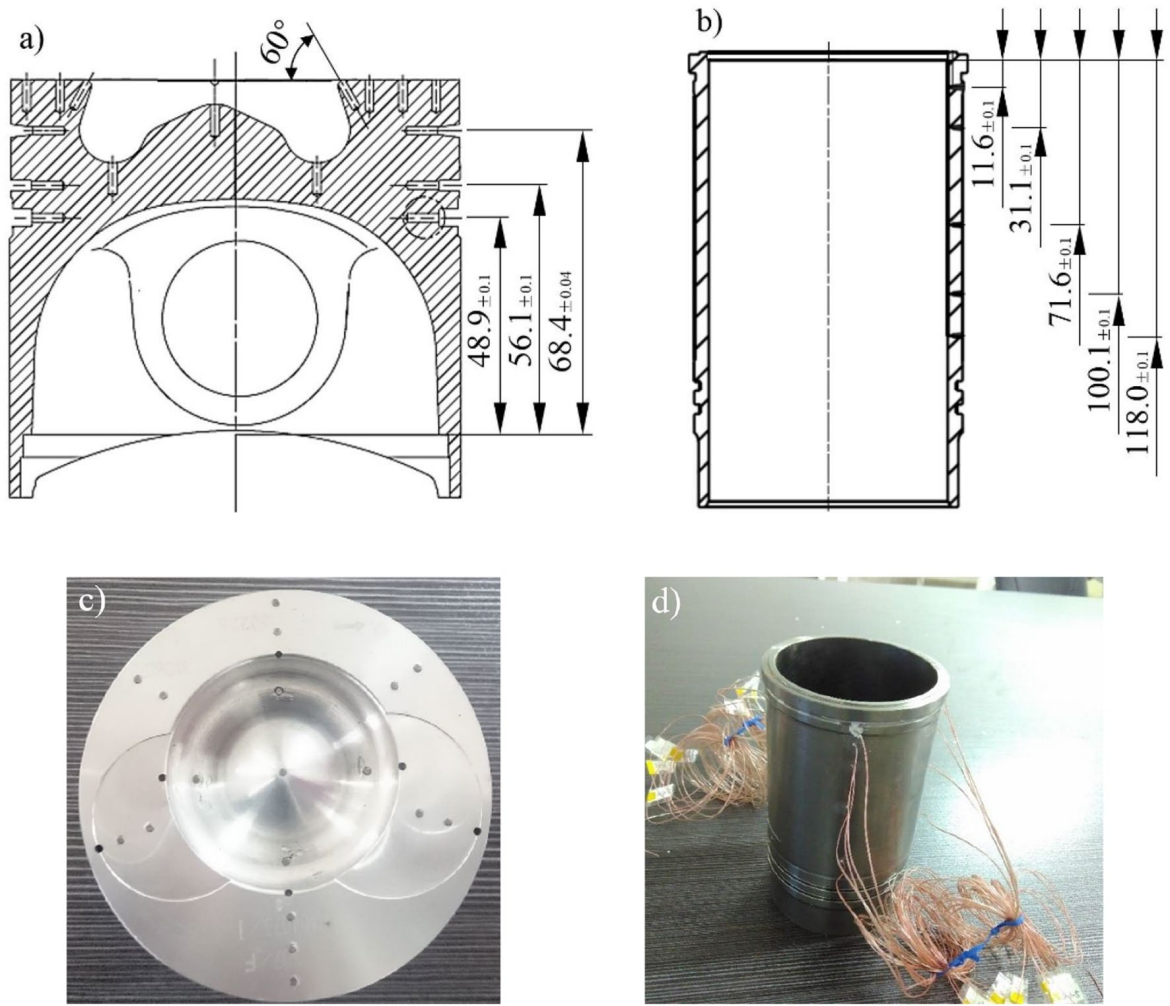
Temperature measure locations distribution of (**a**) piston, and (**b**) liner. Test objects of (**c**) piston, and (**d**) liner.

Hardness plug method was conducted to measure the temperature distribution of piston. Simultaneously, using thermocouple method to measure temperature distribution of liner. The hardness plug is made of GCr15 bearing steel and processed into a Φ1.9 × 5.6 pin. After the test end surface was ground and polished, the hardness of the hardness plug under the same batch of different tempering temperatures was measured and calibrated by the HXD-1000TC micro hardness instrument. Piston temperature measure locations distribution is shown in Fig. 3a. Each location was located every 7.5 mm from the throat position to the outer edge of the piston top surface. 4 measure locations were arranged at the bottom of the combustion chamber, the throat and each ring groove respectively. Piston with plug holes is shown in Fig. 3c.

Omega’s T-type thermocouples were used to measure the temperature of the outer surface of the liner. The measure locations distribution is shown in Fig. 3b. Five rows of measure locations were uniform distributed on the outer surface of the liner at 0°, 90°, 180° and 270°. The five heights of each row correspond to the positions of the first ring at TDC, the oil ring at TDC, the first ring at the half time of the stroke, the first ring at the time of the three-quarters of the stroke, the top surface of the piston at BDC (bottom dead centre). The depth of the measure location holes is 2 mm. Liner with thermocouples is shown in Fig. 3d.

**Figure 4:**
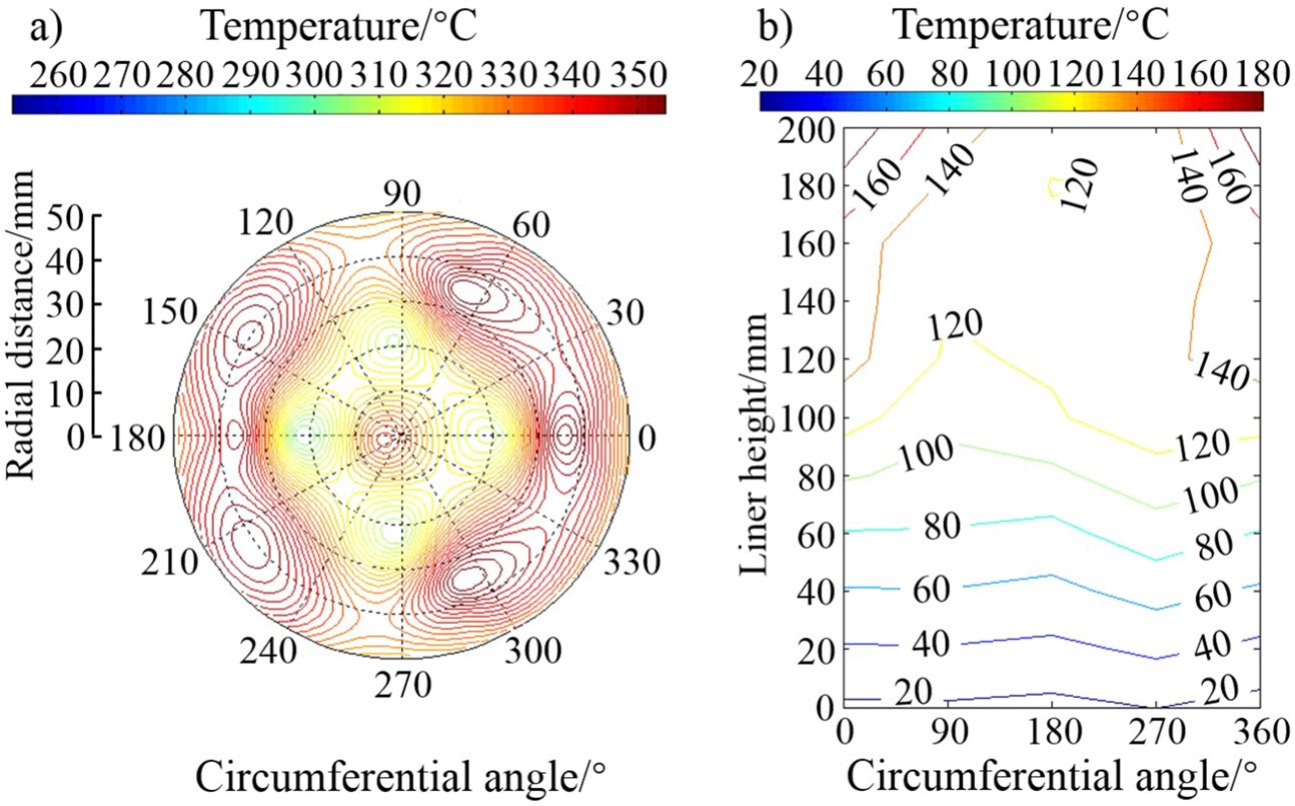
Isothermal distribution of piston and liner temperature field of (**a**) piston top surface, and (**b**) outer surface of the liner.

Engine bench test at room temperature (about 20 °C) was conducted after installing the piston hardness plugs and liner thermocouples. The test process started from low speed and low torque up to calibration condition (2400 r/min, 85 kW) gradually. Temperature data was collected after two-hour stable operation under the calibration condition. The hardness plugs were taken out by wire EDM. After being ground and polished, the hardness value was measured with the micro hardness device to obtain the corresponding temperature results. The distribution of isotherms on the top surface of the piston and the outer surface of the cylinder liner is shown in Fig. 4 by MATLAB analysis.

### Finite-element analysis of temperature field and deformation

Based on the temperature boundary conditions obtained from the test, a steady-state finite element analysis of the temperature field of the piston and liner was conducted. The heat transfer boundary of each area was adjusted many times to correct the model to obtain the temperature field distribution of the piston and liner. The main material properties of piston and liner are listed in Table 2.

**Table 2:** Main material properties of piston and liner.

Parameter	Value	
	Piston (aluminium alloy)	Liner (steel)
Conductivity (kW m^−1^ k^-1^)	140	50.0
Density (kg/m^3^)	2800	7800
Elastic (N/mm^2^)	75,000	210,000
Poisson ratio	0.33	0.3
Expansion (K^−1^)	2.071e−05	1.000e−05

**Figure 5:**
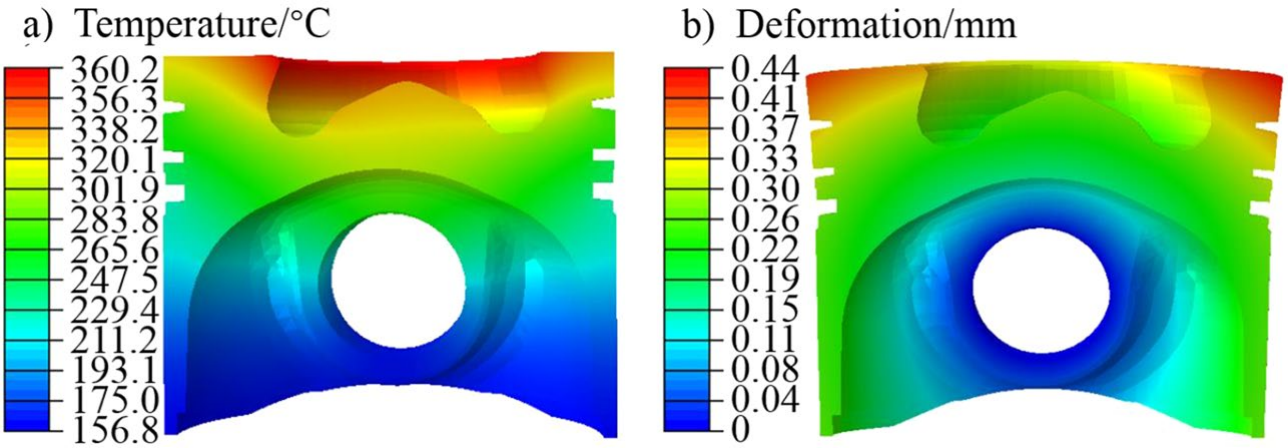
Piston simulation results of (**a**) temperature, and (**b**) deformation.

**Figure 6:**
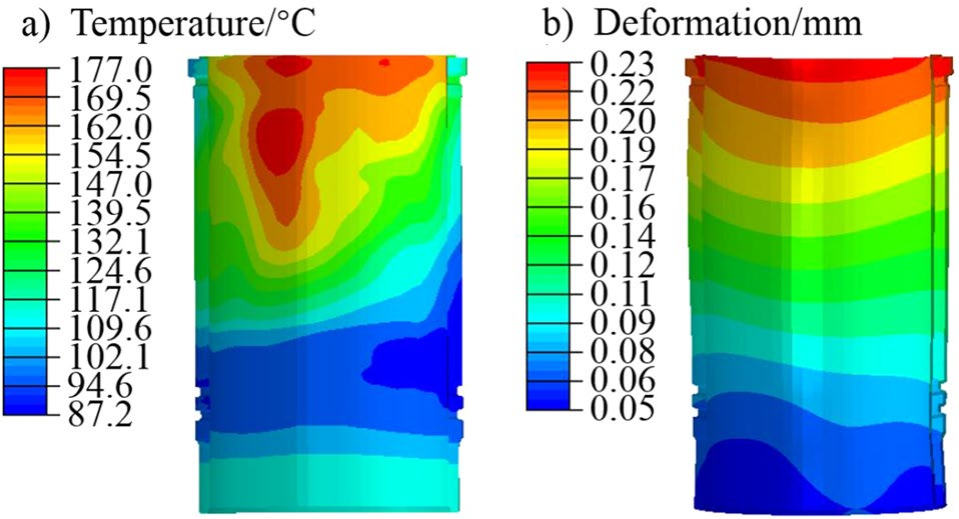
Cylinder liner simulation results of (**a**) temperature, and (**b**) deformation.

The simulation result of the temperature field was used as a predefined temperature field. Gas pressure was applied to the top surface of the piston, while restraining the degree of freedom of the contact area between the piston pin and the small end of the connecting rod. The thermo-mechanical coupling deformation analysis of the piston under peak pressure condition was carried out. The thermal–mechanical coupling deformation analysis of the liner was conducted with the finite element model of the cylinder head-liner-block-bearing cover, cylinder head bolts and main bearing bolts. The analysis combined the temperature field, bolt pre-tightening force and peak pressure. The temperature field and coupling deformation results of the piston and liner are shown in Figs. 5, 6.

It can be seen from Fig. 5 that the temperature of the piston top surface area shows a decreasing trend with the increase of the radius from the combustion chamber throat to the outer edge. The highest temperature of the piston top surface is mainly distributed in the throat area of the combustion chamber, and the highest temperature reaches 360.2 °C, which is also the highest temperature of the piston. Along the axis, the temperature distribution from the top to the bottom of the piston also shows a gradient decreasing trend. The highest temperature in the top land area is about 330 °C, which is located on the upper edge. The thermal–mechanical coupling deformation of the piston indicates a warping deformation trend on the top surface. The maximum deformation is about 0.44 mm.

In order to simulate the deformation of the liner more accurately, the calculation of its deformation took into account the influence of cylinder head bolt pre-tightening force, peak gas pressure and thermal load. In the actual working process of this engine, the thermal load of the third liner was the highest. The calculation results of the third cylinder were used for further analysis. The temperature field and deformation results of the finite element calculation of the liner are shown in Fig. 6. The maximum temperature of the liner is located 18 mm from the top of the TS side, which is 177 °C. The maximum deformation position is about 28 mm from the top of the liner, and the maximum deformation is 0.23 mm.

### Piston assembly dynamic modelling and validation

**Figure 7:**
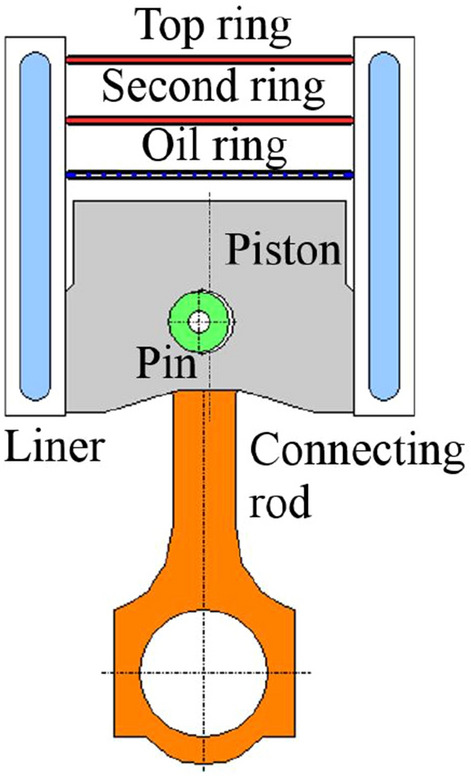
Piston assembly dynamic model.

During the working process of the internal combustion engine, piston and liner will be deformed due to the coupling effect of complex mechanical load and high temperature thermal load. It is difficult to build the secondary motion model of the piston accurately^24,25^. Therefore, it is necessary to make the following assumptions for the simulation model of piston movement: (1) Only the movement of the piston assembly on the plane formed by TS and ATS was considered in the calculation. The slight movement in the direction of the pinhole axis was ignored. (2)The crankshaft was considered to rotate at a constant speed in the calculation. (3) The piston body was set as an elastic body in the calculation. The liner, connecting rod, crankshaft and other components were considered as rigid bodies. The connection gaps were zero. (4) Only the deformation of piston along the radius direction was considered in the calculation. The deformation along the cylinder axis was ignored. The completed dynamic simulation model included piston, pin, connecting rod, piston rings (two gas rings and one oil ring) and liner. Parameters such as in-cylinder pressure, gas temperature and coefficient of heat transfer under calibration conditions were extracted from the one-dimensional simulation model. Piston profile and liner profile under thermal load and pressure are necessary for the calculation. The profiles under loads are the superposition of their original profiles and the thermal–mechanical coupling deformation extracted from the FEM, which were inputted as boundary conditions for dynamic calculation. The dynamic model is shown in Fig. 7.

**Figure 8:**
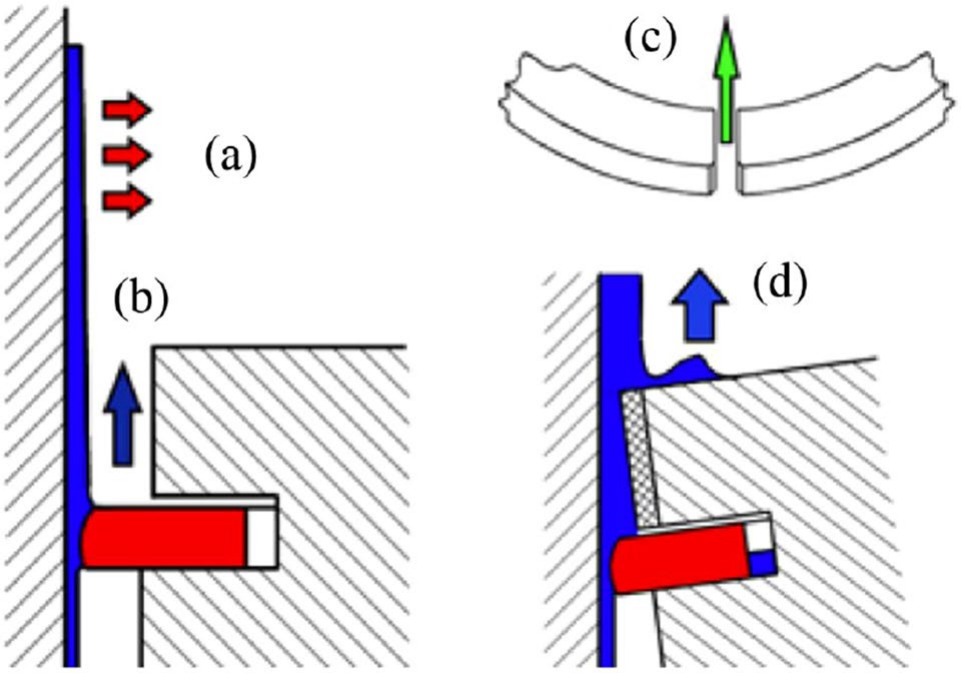
Lubricating oil consumption paths of (**a**) evaporation from liner wall, (**b**) oil throw off, (**c**) oil blow through top ring end gap, (**d**) oil scrapping of the top land.

Oil consumption was the model verification indicator for test verification. It mainly includes the following four paths: (1) The remaining lubricating oil on the liner wall evaporated by the high-temperature gas. (2) The lubricating oil accumulated on the upper surface of the first piston ring thrown into combustion chamber under the action of inertial force. (3) When a negative pressure gradient occurs (the gas pressure in the combustion chamber is lower than the pressure at the land between the first ring and the second ring), the lubricating oil is brought into the combustion chamber by the gas through the end gap of the first piston ring. (4) Oil scraping at the top land of the piston. The schematic diagrams of the four oil consumption paths are shown in Fig. 8. The simulation calculation results are derived from the distribution of temperature, pressure, oil, etc. of the contact surfaces of the piston, rings, and liner in each working cycle. Therefore, oil consumption is considered to be the model verification parameter which could comprehensively verify the accuracy of the dynamics model.

Before the test, the weight of the oil in the oil sump was measured by the oil drain weighing method. After weighing, the oil was poured back into the oil sump to start the test. The oil was drained again after a 6-h stable operating of the diesel engine under the calibration condition to obtain the oil consumption rate. The test was repeated three times, and the comparison between the test results and the simulation result of the model built above is shown in Table 3. The deviation between the oil consumption simulation result and the average test result is 5.14%. Thus, it can be assumed that the simulation model was accurate for subsequent dynamic analysis.

**Table 3:** Oil consumption results.

Item	Value
1st test (g/kW h)	0.232
2nd (g/kW h)	0.272
3rd (g/kW h)	0.254
Test average (g/kW h)	0.253
Simulation (g/kW h)	0.266
Deviation (%)	5.14

## Results and discussion

### Effects of piston assembly key structural parameters on secondary motion characteristics

**Figure 9:**
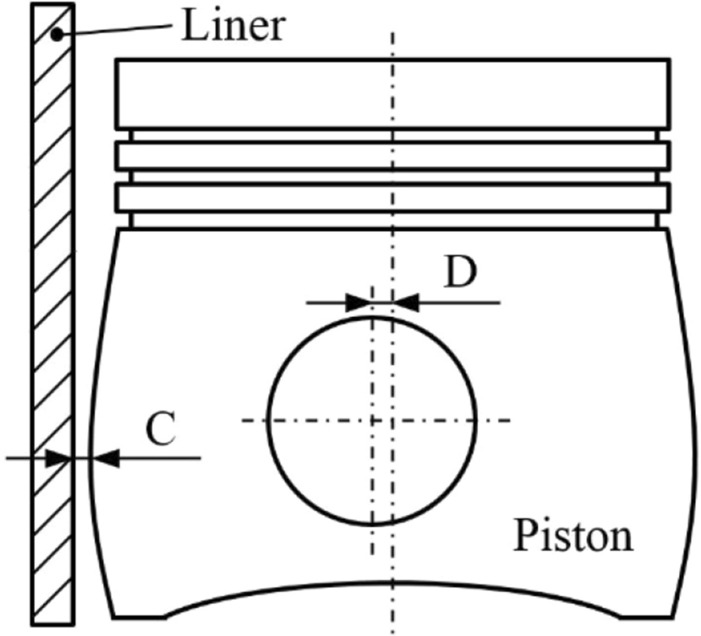
Schematic of pinhole offset (D) and cylinder clearance (C).

Studies have shown that there are many factors affecting the secondary motion of the piston^26^. These factors are categorized as external factors and internal factors. As far as external factors are concerned, the engine speed and load have a great influence on the secondary motion of the piston. As far as internal factors are concerned, the structural parameters of the piston, the characteristics of the piston-rings-liner friction pair, the piston skirt profile and other parameters affect a lot on the piston motion law. The offset design of the piston pin is beneficial to reduce the maximum slapping energy of the piston and reduce the impact noise. However, it would cause an adverse effect on the friction and wear of the skirt at the same time^27^. The liner clearance and the ovality of the skirt are also important influence factors of skirt friction. When the liner clearance is too large, it would greatly increase the slapping energy between the piston and the liner. However, if it is too small, the wear between the piston and the liner would increase, which may even cause severe liner scuffing. An appropriate ovality design can make the piston deform into a reasonable pressure-bearing surface through thermal–mechanical coupling load under working conditions, which will improve the lubrication characteristics of the piston-rings-liner friction pair. Pinhole offset (D) and cylinder clearance (C) are shown in Fig. 9.

The secondary motion of the piston is generally illustrated by the radial displacement and the tilting angle of the piston. The slapping energy of the piston can directly reflect the roughness of piston reverse. The friction power loss directly reflects the friction performance of the piston skirt. Both of the above are important indicators of piston secondary motion characteristics. This chapter analyses the relationship between three key structural parameters and the secondary motion of the piston based on the established piston assembly dynamic model.

#### Effects of pinhole offset

**Figure 10:**
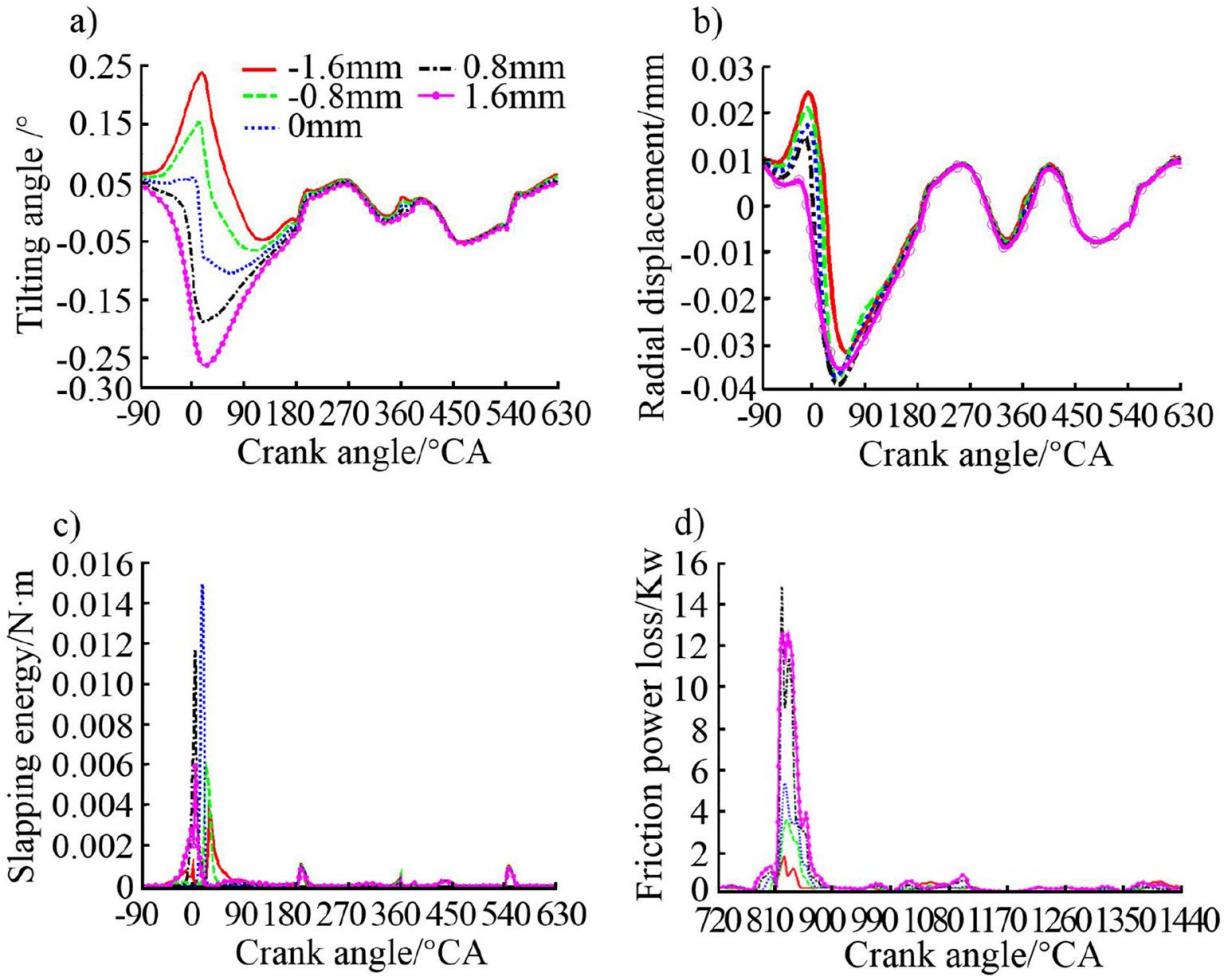
Effect of piston pinhole offset on (**a**) piston tilting angle, (**b**) piston radial displacement, (**c**) piston slapping energy, and (**d**) piston friction power loss.

The purpose of the asymmetric design of the piston with pinhole offset is to apply a torque to the piston to prevent it from suddenly moving from the ATS to the TS after TDC and producing slapping noise. In order to study the effect of the pinhole offset on the secondary motion characteristics of the piston, five groups of offset schemes including − 1.6 mm, − 0.8 mm, 0 mm, 0.8 mm, and 1.6 mm were chosen for research where the negative sign is biased to the TS. The five values were chosen under the premise of ensuring the normal operation of the piston. Figure 10 shows the variation of four performance characteristics of tilting angle, radial displacement, slapping energy, and friction power loss with the crankshaft angle in a working cycle.

It can be seen from Fig. 10 that the piston pinhole offset has a great impact on the secondary motion characteristics of the piston, especially the tilting angle of the piston. Figure 10a shows that the pinhole offset has the most significant effect on the tilting angle of the piston during the compression stroke and expansion stroke, especially at the TDC. The effect of the offset direction of the pinhole is also very significant. The piston tilting angle is the smallest when the pinhole is not offset. In Fig. 10c, it can be seen that no matter whether the pinhole is biased to the TS or the ATS, it can reduce the sudden slapping caused by the side force at the TDC when the piston reverses from the ATS to the TS. The impact moment of the peak pressure is delayed when the hole is biased to the TS.

The analysis shows that the piston moves up against the ATS during the compression stroke when the pinhole is biased to the TS. As the in-cylinder pressure increases, the gas pressure acts on the top surface of the piston to produce a moment of rotation around the pin. The moment causes the piston to rotate before reaching the TDC. The lower part of the skirt on the TS contacts the cylinder liner in advance, and then the contact area transfers to the upper part of the skirt after reverse, thus avoiding the reverse at the peak pressure to reduce the slapping energy. When the pinhole is biased to the ATS, the reverse effect caused by the gas pressure is that the upper part of the piston skirt with greater stiffness contacts the liner first, which will cause more severe slapping. It can be seen from Fig. 10c that the peak value of the slapping energy of the ATS biased schemes is slightly larger than that of the TS biased schemes. Figure 10d shows that the peak value of friction power loss always appears in the work stroke, and then fluctuates in a small range. The pinhole offset value and the total cyclic friction power loss show an obvious linear relationship. The total friction loss gradually increases with the piston pinhole moves from the TS to the ATS.

#### Effects of liner clearance

**Figure 11:**
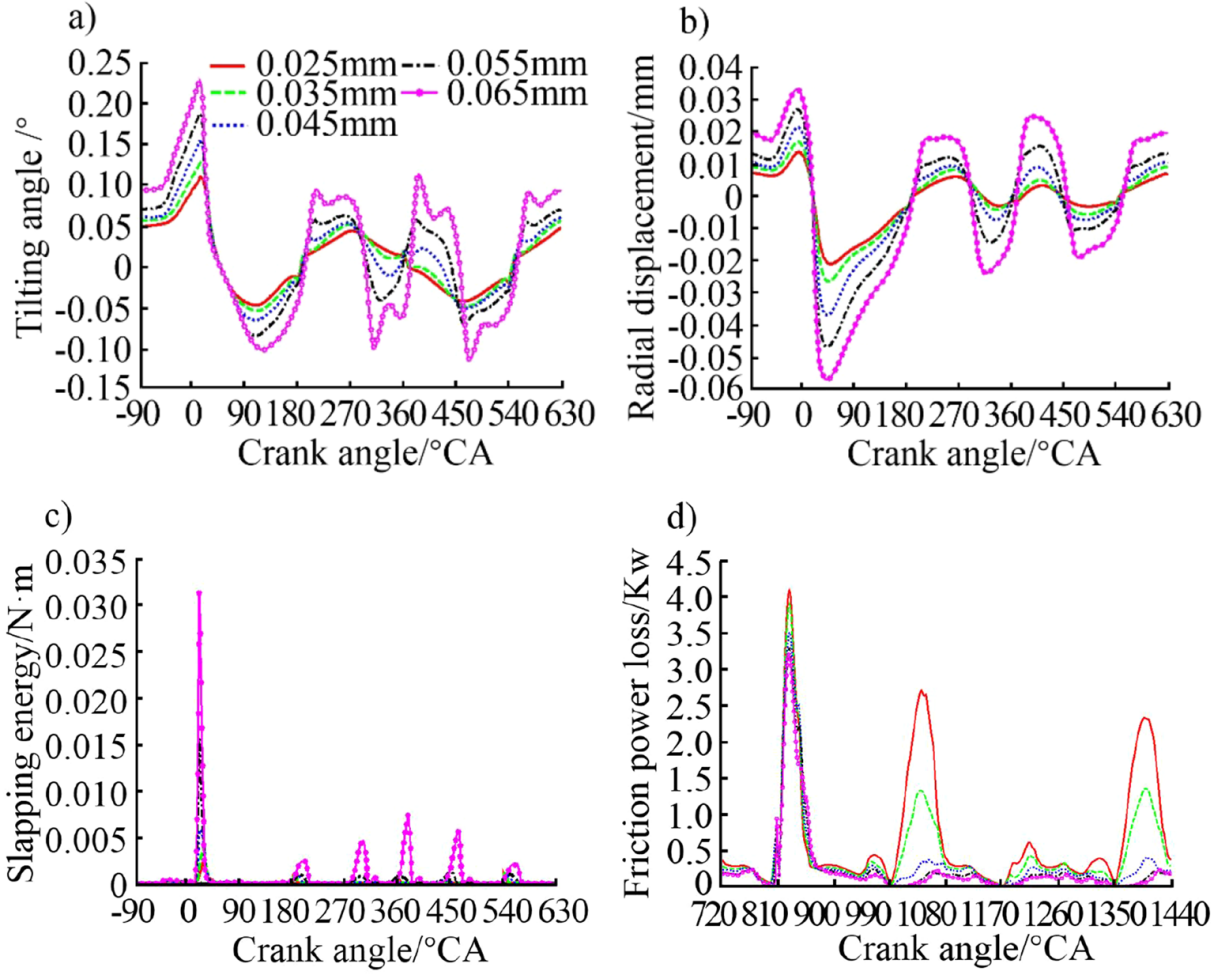
Effect of liner clearance on (**a**) piston tilting angle, (**b**) piston radial displacement, (**c**) piston slapping energy, and (**d**) piston friction power loss.

The design of the liner clearance has a significant impact on the performance of the internal combustion engine. The design should fully consider the load and speed of the skirt to ensure sufficient lubrication of the piston skirt. The clearance after the piston is heated and deformed should also be considered. Therefore, the liner clearance value should not be too small in order to avoid liner scuffing during the working conditions. Certainly not too large, so as to reduce the slapping energy and noise. In order to analyse the effect of the liner clearance on the secondary motion of the piston, five schemes of clearances including 0.025 mm, 0.035 mm, 0.045 mm, 0.055 mm, and 0.065 mm were set for simulation. The five values were chosen under the premise of ensuring the normal operation of the piston. The calculation results are shown in Fig. 11.

It can be seen from Fig. 11a,b that the piston tilting angle and radial displacement will increase correspondingly with the increase of the liner clearance. The degree of piston tilting varies obviously with different liner clearances, but the trend during a working cycle is the same. It is analysed that with the increase of the liner clearance, the offset of each point on the piston from the liner increases, which will inevitably cause the increase of the radial displacement and lead to the deterioration of the secondary motion characteristics of the piston. Figure 11c shows that the liner clearance has a significant impact on the slapping energy of the piston. The energy is 0.00236 N m when the clearance is 0.025 mm, which increases to 0.0314 N m when the clearance is 0.065 mm, that is, an increase of more than 13 times. Therefore, increasing the liner clearance will cause the piston to increase its tilting during operation, thereby deteriorating the slapping and NVH performance of the internal combustion engine. The reduction of the liner clearance can stabilize the reciprocating movement of the piston. However, the contact stress between the piston and the liner will increase if the clearance is too small so that it will be difficult to form a sufficient lubricating oil film, resulting in deterioration of friction and wear and even liner scuffing. The difference in friction power loss in the work stroke under different liner clearance is small, and the difference in exhaust and compression strokes is large. The friction power loss throughout the cycle decreases from 0.69 to 0.24 kW with the increase of liner clearance from 0.025 mm to 0.065 mm. It is analysed that a small liner clearance is not beneficial to the formation and maintenance of lubricating oil film, resulting in increased friction power loss.

#### Effects of skirt ovality

**Figure 12:**
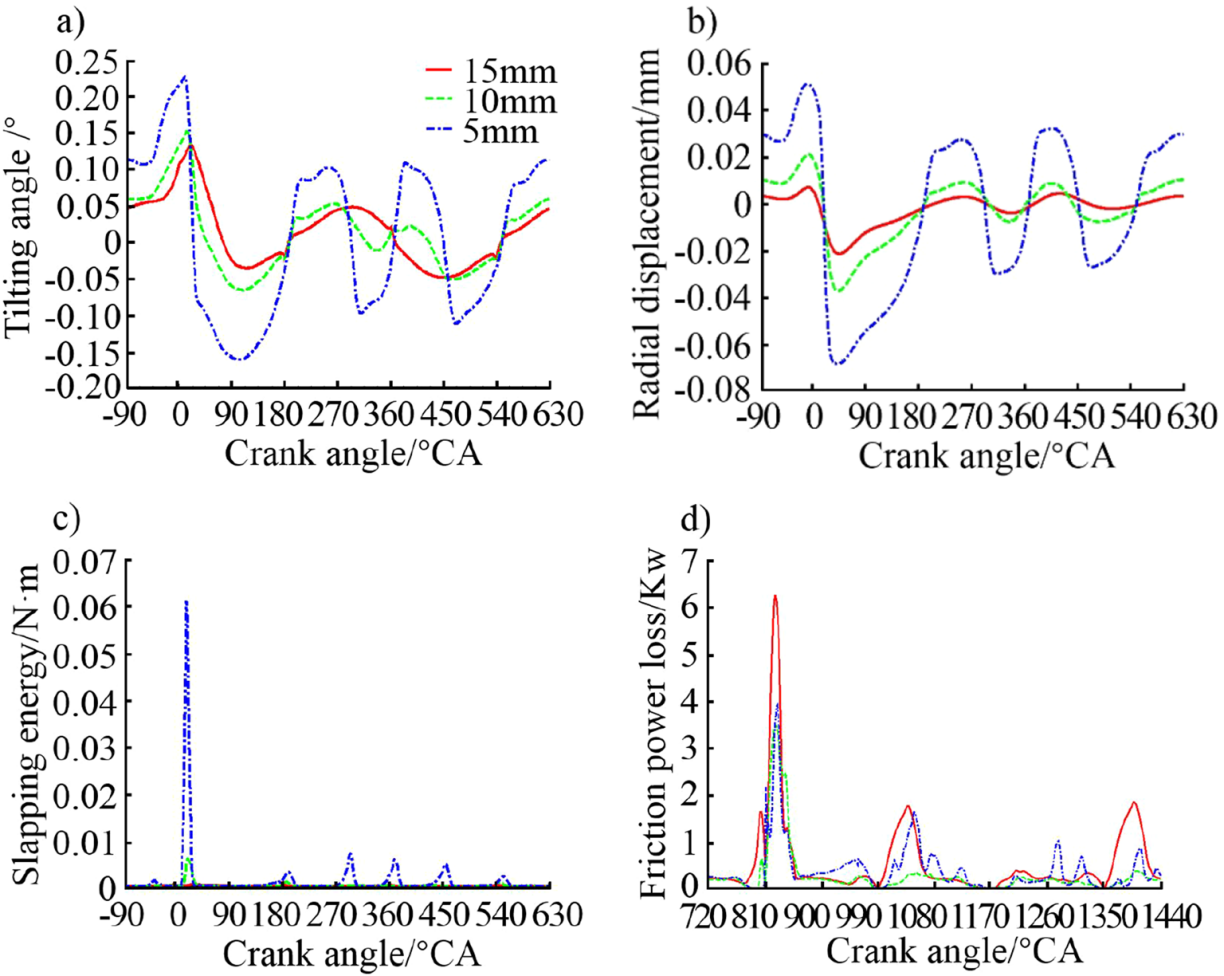
Effect of skirt ovality on (**a**) piston tilting angle, (**b**) piston radial displacement, (**c**) piston slapping energy, and (**d**) piston friction power loss.

The outer edge of cross section of the piston skirt is generally elliptical in the piston design nowadays, rather than a perfect circle, which was widely used during the early years. The elliptical cross section can improve the contact between the piston and the liner under working conditions, and has a significant impact on the performance of the internal combustion engine. Three ovality values including 0.2 mm, 0.4 mm (original size) and 0.6 mm were set on the piston skirt for dynamic simulation analysis. Figure 12 shows the effect of different ovality on the secondary motion of the piston and the slapping energy of piston slapping and friction power loss.

It can be seen from Fig. 12 that as the skirt ovality increases, the piston tilting angle, radial displacement and slapping energy increase. While the friction power loss decreases with the increase of the ovality. It is analysed that a large value of ovality leads to a reduction in the contact area between the piston skirt (especially the TS) and the liner, which reduces the restriction on the secondary motion of the piston. On the one hand, tilting angle, radial displacement and slapping energy increase as a result. On the other hand, a reduction in the contact area will result in a reduction in friction loss. However, a too small contact area will adversely affect the lubrication between the skirt and the liner. Therefore, a reasonable design of the ovality of the piston skirt can match with the thermal–mechanical coupling deformation of the piston under working conditions. In that way a good piston-liner contact pressure surface would be formed, thereby improving the secondary motion characteristics and optimizing the friction and lubrication performance of the piston assembly.

### Multi-factor analysis of piston secondary motion characteristics based on response surface methodology

The previous chapters mainly analysed the single factor effect on the secondary motion characteristics of the piston assembly. However, the influence of different combinations of factors will have an interactive effect on the dynamic characteristics. Therefore, it is necessary to calculate and analyse different combination schemes’ effects.

#### Response surface modelling

Using the response surface methodology, the pinhole offset, the liner clearance and the skirt ovality were defined as factors A, B and C respectively. Three levels were set for each factor respectively. The friction power loss and the slapping energy were set as evaluation indicators and defined as V_1_ and V_2_ response respectively. The experiment was designed based on Box–Behnken method. Established the model between responses and factors. The level values and simulation results of each plan are shown in Table 4. The regression models for the two responses are as follows:

4 $$\begin{aligned} V &_{1} = - 0.361 + 0.092A + 14.865B + 3.389C - 0.175AB \\ & \quad + 0.222AC - 54.650BC + 0.001A^{2} + 7.000B^{2} - 0.341C^{2} \\ \end{aligned}$$

5 $$\begin{aligned} V_{2} & = 0.010 + 0.005A + 1.435B + 0.059C - 0.386AB \\ & \quad - 0.007AC - 1.15BC - 0.003A^{2} + 33.511B^{2} - 0.342C^{2} \\ \end{aligned}$$

**Table 4:** Response surface factor values and simulation results.

Calculation plan	Pinhole offset A/mm	Liner clearance B/mm	Skirt ovality C/mm	Friction power loss *V*_1_/kW	Slapping energy *V*_2_/N m
1	− 0.8	0.02	0.4	0.942	0.00424
2	0.8	0.02	0.4	0.385	0.00180
3	− 0.8	0.07	0.4	0.611	0.07102
4	0.8	0.07	0.4	0.582	0.00065
5	− 0.8	0.04	0.2	0.932	0.00987
6	0.8	0.04	0.2	1.195	0.00183
7	− 0.8	0.04	0.6	0.639	0.01108
8	0.8	0.04	0.6	0.599	0.01660
9	0	0.02	0.2	0.353	0.04012
10	0	0.07	0.2	0.619	0.07401
11	0	0.02	0.6	0.662	0.00120
12	0	0.07	0.6	0.328	0.04802
13	0	0.04	0.4	0.391	0.00320

The significance test of the above models was carried out^28^. The model’s determination coefficient $R^{2}$ and adjustment determination coefficient $R_{adj}^{2}$ were used to evaluate the fitting degree of the model. Generally, both coefficients are required to be greater than 0.9. The test results show that the two coefficients of the *V*_1_ model are 0.999 and 0.998, and those of the *V*_2_ model are 0.994 and 0.975. Therefore, it is believed that the two response models can fit the results of the simulation test accurately, and can be used for further analysis and prediction of friction power loss and slapping energy. The significance test results of the regression coefficient are shown in Table 5. *F*_1_ and *p*_1_ are the *F* value and *p* value corresponding to the *V*_1_ response respectively, so are *F*_2_ and *p*_2_. It is generally believed that the factor has a significant meaning for the response if the *p* value of it is less than 0.05. The larger the *F* value and the smaller the *p* value are, the more significant the factor effect is.

**Table 5:** Significance test of regression models.

Item	*F*_1_	*P*_1_	*F*_2_	*P*_2_
A	15625.85	< 0.0001	24.36	0.0160
B	20776.01	< 0.0001	344.88	0.0003
C	14161.55	< 0.0001	7.15	0.0755
AB	5.03	0.1108	13.07	0.0365
AC	517.03	0.0002	0.24	0.6577
BC	30632.03	< 0.0001	7.29	0.0738
A^2^	0.09	0.7820	0.57	0.5044
B^2^	4.49	0.1244	55.11	0.0051
C^2^	43.52	0.0071	0.23	0.6614

#### Multi-factor response surface analysis of friction power loss

According to the test results of the regression coefficients of friction power loss in Table 4, the primary terms of each factor in the model are statistically significant. The significance order of the three factors can be seen as follows by comparing the F value: liner clearance > pinhole offset > skirt ovality. The interaction items of pinhole offset and skirt ovality, and liner clearance and skirt ovality are significant. The others are not significant or have a much lower significance than the above items. The surface response diagrams of friction power loss under the interaction of each combination are as follows.

**Figure 13:**
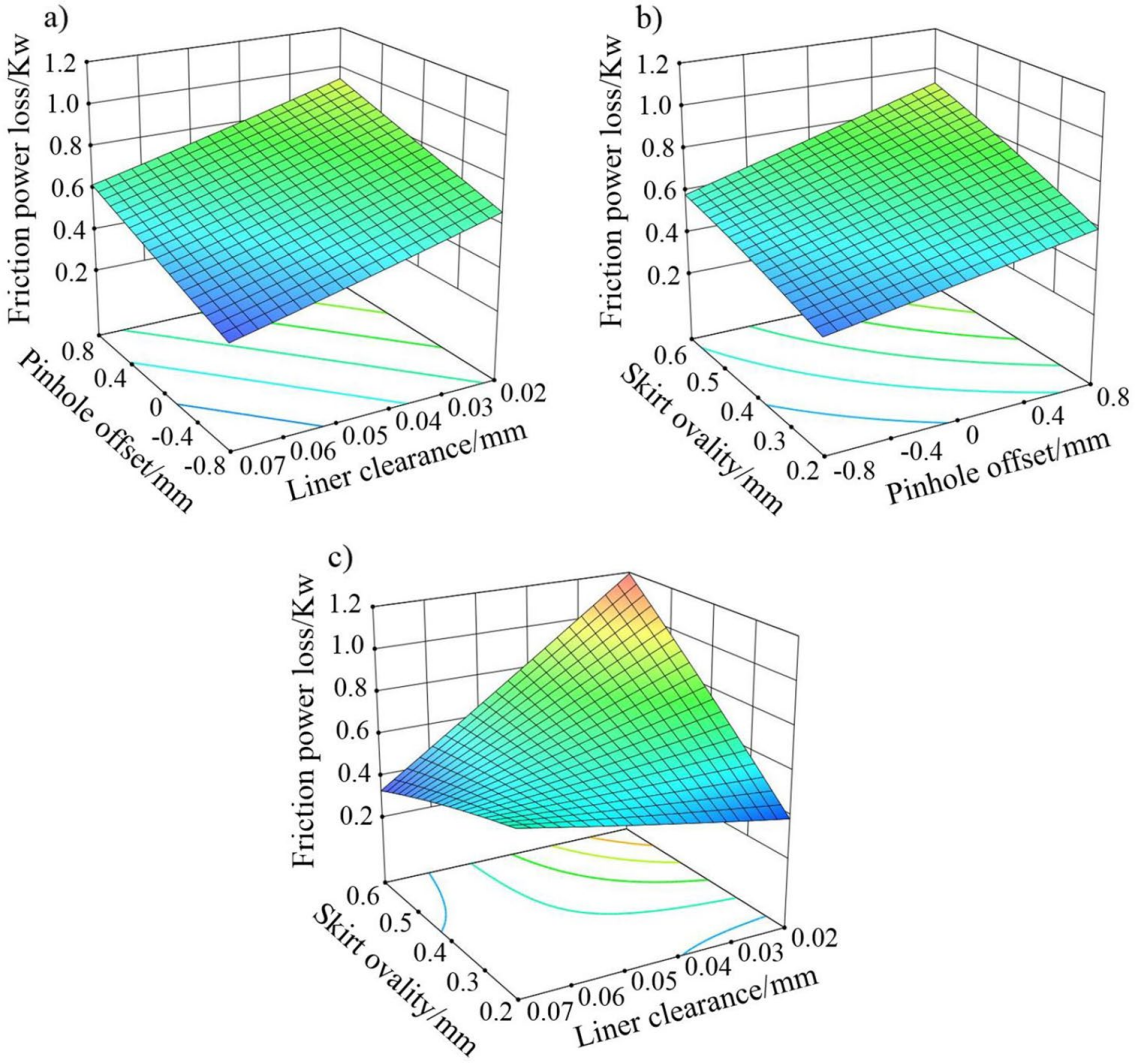
Response surfaces of effects on friction power loss of (**a**) pinhole offset and liner clearance, (**b**) pinhole offset and skirt ovality, and (**c**) liner clearance and skirt ovality.

It can be seen from Fig. 13a that the interaction influence of pinhole offset and liner clearance on friction power loss shows an obvious linear relationship under a constant skirt ovality. Under the condition of a fixed liner clearance, the friction power loss has a positive correlation with the pinhole offset. While the pinhole offset remains unchanged, the friction power loss has a negative correlation with the liner clearance. The contour plot of the surface projection is basically made up of straight parallel lines. Figure 13b indicates that, in the case of a constant liner clearance, the influence of pinhole offset and skirt ovality on friction power loss shows a linear relationship to a certain extent. When the pinhole offset is also constant, the friction power loss is positively related to the skirt ovality. The skirt ovality has a larger influence on the friction loss when the pinhole is offset to the ATS. While the ovality is constant, the friction power loss is also positively correlated to the pinhole offset, and the minimum value appears when the skirt ovality and pinhole offset are both at their minimum. As far as a constant pinhole offset is concerned, Fig. 13c indicates that the interaction effect of the liner clearance and skirt ovality on the friction power loss is not an obvious linear relationship. The combinations of small skirt ovality/small liner clearance and big skirt ovality/big liner clearance result in a lower friction power loss. While small skirt ovality/big liner clearance combination leads to a higher friction power loss, and big skirt ovality/small liner clearance leads to the highest. The analysis demonstrates that when the skirt ovality is relatively consistent with the selection trend of the liner clearance, the piston and the liner match well, especially under the thermal–mechanical coupling deformation during the working process. Thereby the lubrication state of the skirt is improved and the friction loss is reduced.

#### Multi-factor response surface analysis of slapping energy

According to the significance test of the regression coefficient in Table 4, it can be seen that the significant factors on the slapping energy include pinhole offset, liner clearance (and the square of it) and the interaction between the two. The rest are not significant. The most significant factor is the liner clearance by comparing the F value. The surface response diagrams of slapping energy under the interaction of each combination are as follows.

**Figure 14:**
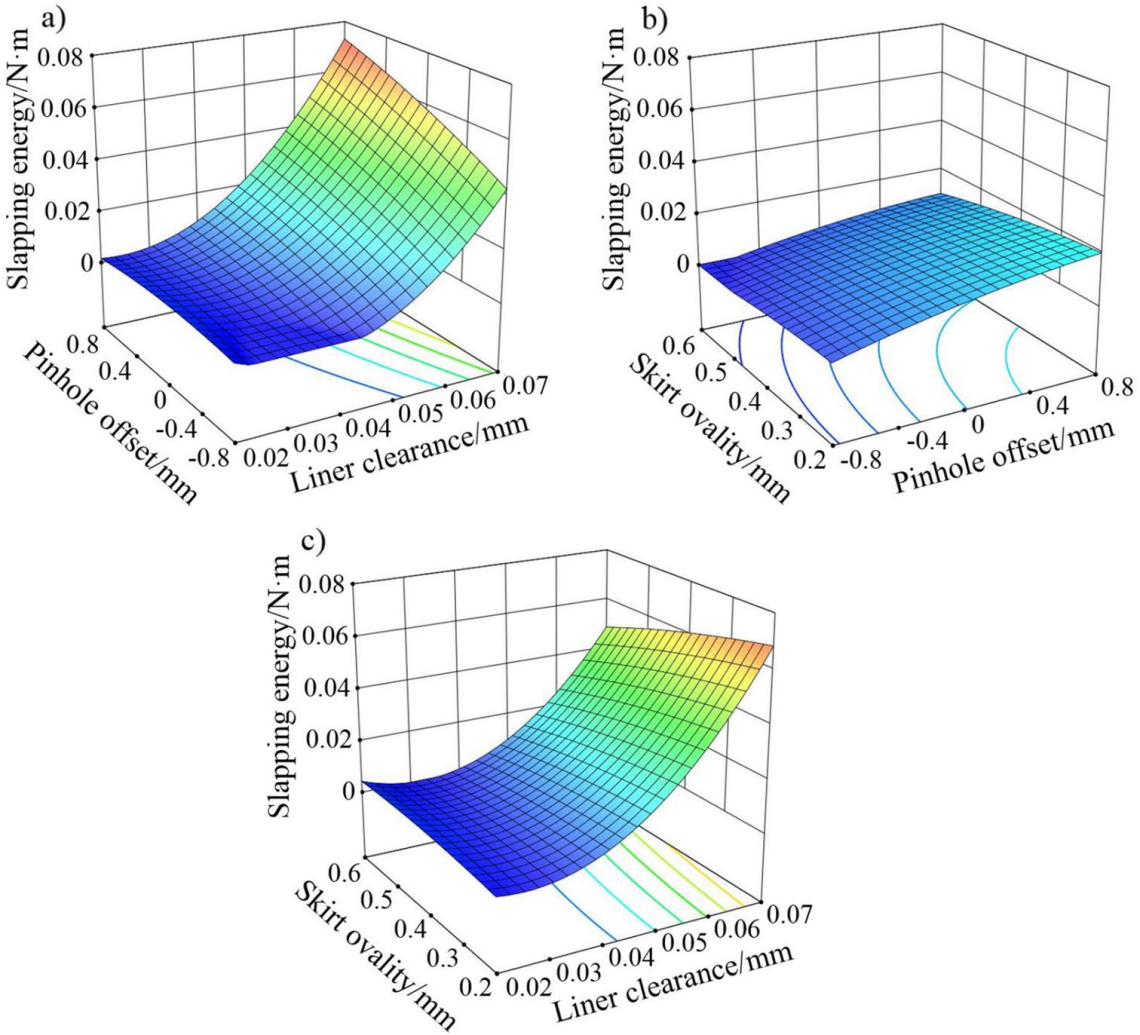
Response surfaces of effects on slapping energy of (**a**) pinhole offset and liner clearance, (**b**) pinhole offset and skirt ovality, and (**c**) liner clearance and skirt ovality.

It can be seen from Fig. 14a that under the condition of a constant skirt ovality, the slapping energy is at a low level under small liner clearance but high under big liner clearance. The impact of the pinhole offset on the slapping energy gradually increases while the offset moves from the TS to the ATS. According to Fig. 14b, the slapping energy is positively correlated with the pinhole offset and negatively correlated with the skirt ovality under the condition of constant liner clearance. However, the interaction between the two is not obvious to the slapping energy and the influence range is narrow. Figure 14c shows that, the interaction effect trend of the liner clearance and the skirt ovality on slapping energy is similar to Fig. 14a when the pinhole offset remains unchanged, and the significance of the skirt ovality is negatively correlated with its own value. It is obviously that the liner clearance directly affects the roughness of the piston reverse, so it has the most significant effect on the slapping energy. While according to Fig. 10c, the moment of the peak pressure can be delayed when the pinhole is offset to the TS. Thereby, the pinhole offset and the slapping energy also show a positive correlation to a certain extent.

## Conclusions

On the basis of the conducted research on the relationship between the key structural parameters of the piston and its secondary motion, one can draw the following conclusions:

1. With the pinhole offset changes from 0 to − 1.6 mm or 1.6 mm, the piston tilting angle and radial displacement gradually increase, while the slapping energy can be reduced. Friction power loss increases with the pinhole offset moving from TS to ATS.
2. As the liner clearance increases from 0.025 to 0.065 mm, the tilting angle and the slapping energy of the piston increases significantly with a maximum increase of more than 13 times. The friction power loss decreases for 65% with the increase of liner clearance.
3. The contact area between the skirt and the liner shrinks with the skirt ovality increases from 0.2 to 0.6 mm, resulting in an increase of the tilting angle, radial displacement and slapping energy of the piston. The friction power loss decreases in the meanwhile.
4. Different values of key structural parameters and their combinations have different effects on friction power loss and slapping energy. The pinhole offset, the liner clearance, the skirt ovality and the interaction of the latter two have a significant effect on the friction power loss. While the slapping energy is significantly affected by the liner clearance. The factor value can be designed according to its significance, so as to reduce the friction power loss and the slapping energy of the piston assembly and improve the engine NVH performance.
